# A Method of External Fixation to Offload and Protect the Foot Following Reconstruction in High-Risk Patients: The SALSAstand

**Published:** 2009-06-04

**Authors:** Janice Clark, Joseph L. Mills, David G. Armstrong

**Affiliations:** Southern Arizona Limb Salvage Alliance (SALSA), Department of Surgery, University of Arizona College of Medicine, 1501 N Campbell Ave, Tucson, AZ 85724

## Abstract

**Introduction:** The course of wound healing in high-risk patients with diabetes, particularly those with peripheral arterial disease and renal failure, is often prolonged and fraught with complications. Traditional methods of offloading the posterior foot or holding correction in place following diabetic foot reconstruction include various padded and bolstering devices. **Methods:** In this article, we describe a method (SALSAstand) to effectively elevate, offload, and protect the foot with an external fixation device, while also promoting flap healing, maintaining tendon correction, and limiting the tendon retraction and contracture that is commonly seen following a foot-salvage procedure in high-risk patients. **Results:** Not applicable. **Discussion:** The SALSAstand device has been successfully utilized on many patients in our service to accomplish the aforementioned goals in this most challenging patient population.

Diabetic foot wounds and resultant complications are common, complex, and costly.^1^,^2^ While neuropathic wounds are inherently difficult to heal and keep healed, those compounded by arterial occlusive disease and end-stage renal failure prove in many cases to be clinically refractory, resulting in failure to heal, infection, and high-level amputation.^3^,^4^ In addition, these patients may be at higher risk to develop pressure ulcers of the heel, which now have a minimal acute care incidence of 7% and costs in excess of $40,000 per event. In the United States, recent legislation have made the prevention of these wounds as well as of infections developed during wound care while in hospital a priority, placing the fiscal onus for prevention on the hospital itself.^5^,^6^

The goal of limb-salvage efforts in patients with diabetes is to preserve limb length and function. The use of external fixators to offload heel wounds has been previously reported.^7^,^8^ Castro-Aragon et al^9^ most recently described the construction of an external fixator to prevent heel decubitus ulcer formation in trauma patients. We propose a novel technique to completely offload the heel while providing rapid 3-plane correction and easy access for dressing changes following forefoot amputation or reconstruction in patients at increased risk for more proximal limb amputation.

## METHODS

### Description of procedure

The kickstand device is applied after the foot reconstructive procedure, forefoot amputation (transmetatarsal), foot debridement, or skin graft has been completed. The optimal patient position for the application of the kickstand device is supine.

1. Two half-pins are inserted into the distal one quarter of the tibial shaft along with a rod to the pin connector and the clamp.
2. A transfixion pin is inserted in the foot from medial to lateral through the central portion of the first metatarsal shaft extending laterally through the central portion of the fifth metatarsal shaft.
3. Application of the pin to rod connectors follows.
4. Appropriate foot position in the frontal and sagittal planes is achieved by maneuvering the transfixion pins as handlebars. This is fixed in place if the ideal position is with the foot rectus, without inversion or eversion, and 90° in relation to the lower leg.
5. The appropriate rod length is determined at this time.
6. With the foot in corrected position, apply 1 rod from the distal tibial to the connector located on the medial side of the first metatarsal.
7. Next apply another rod of appropriate length from the tibial to the lateral side of the fifth metatarsal.
8. Assess the position of the foot relative to the lower leg.
9. Apply a rod from the pin on the lateral fifth metatarsal extending inferiorly toward the heel. This will effectively “elevate” the heel off the table. Apply connector at the inferior aspect of this rod.
10. Apply another rod to the medial pin at the first metatarsal, which will extend inferiorly toward the heel. Apply connector at the inferior aspect of this rod.
11. Apply a rod to cross the inferior aspect from medial to lateral rods.
12. Apply rods from the inferior heelposts to the tibial connector.
13. Adjust as necessary.

Postoperative care during the placement should include appropriate pain management, non–weight-bearing wheel chair usage, meticulous pin site care, and antibiotic administration, as indicated.

## CASE EXAMPLES

### Case 1

**Figure 1:**
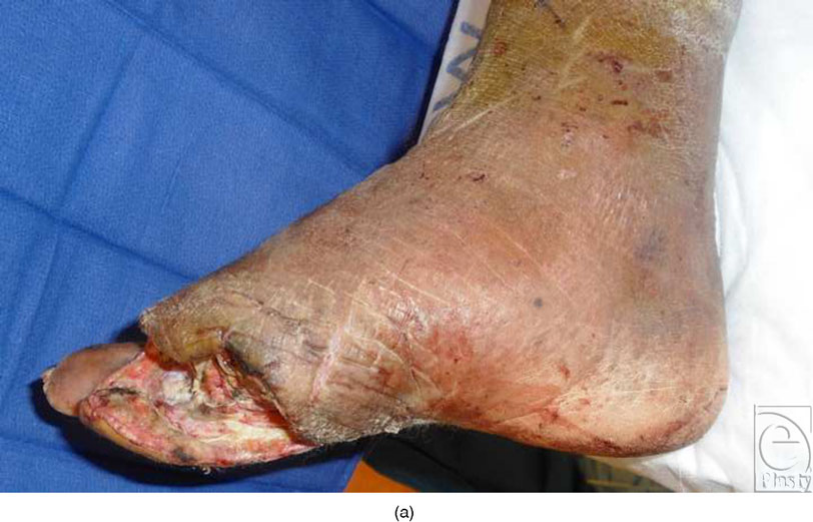

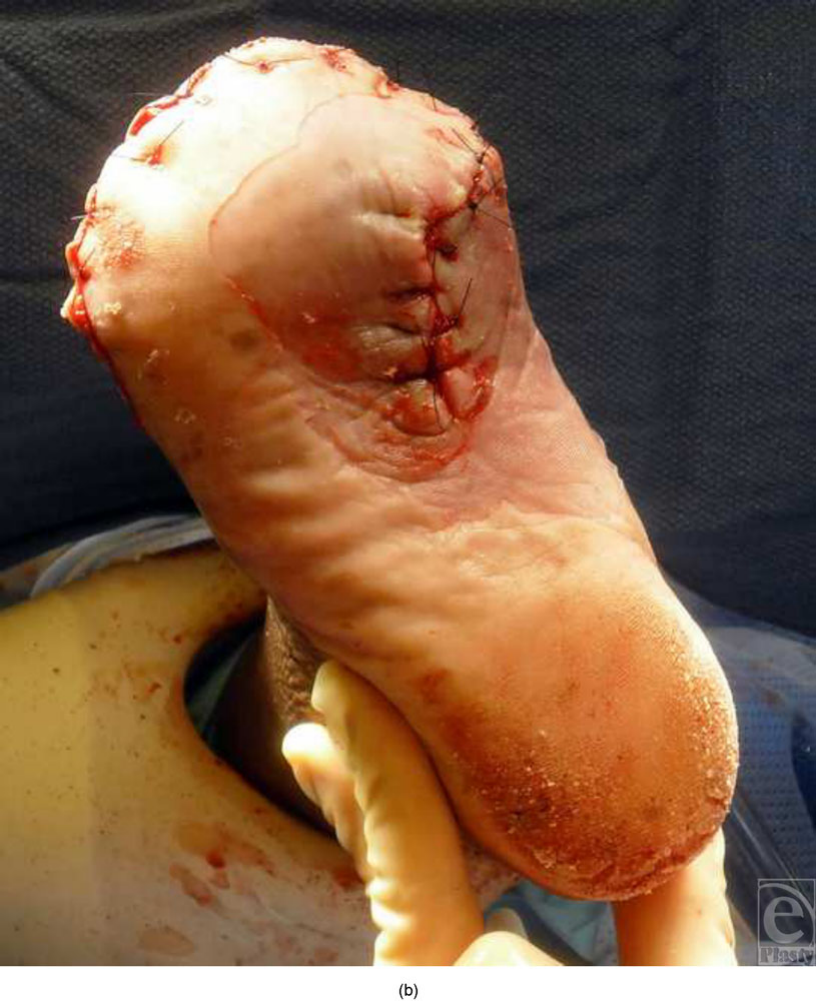

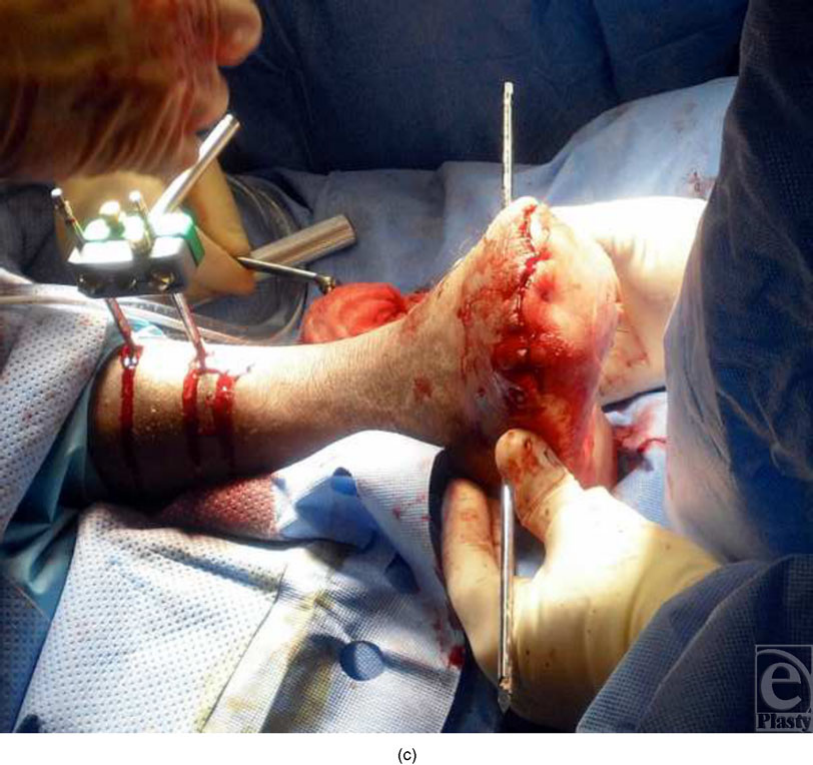

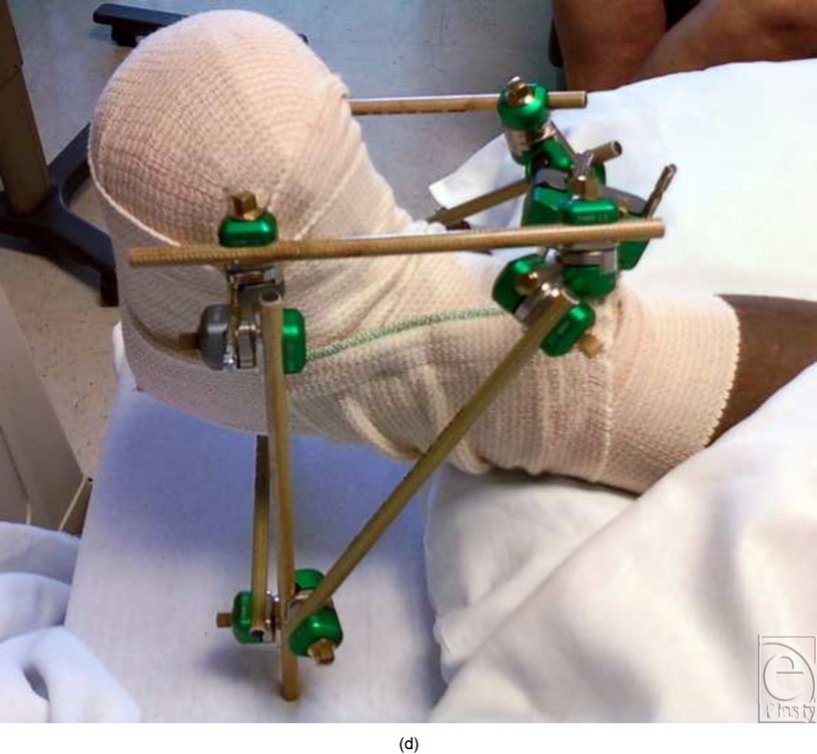

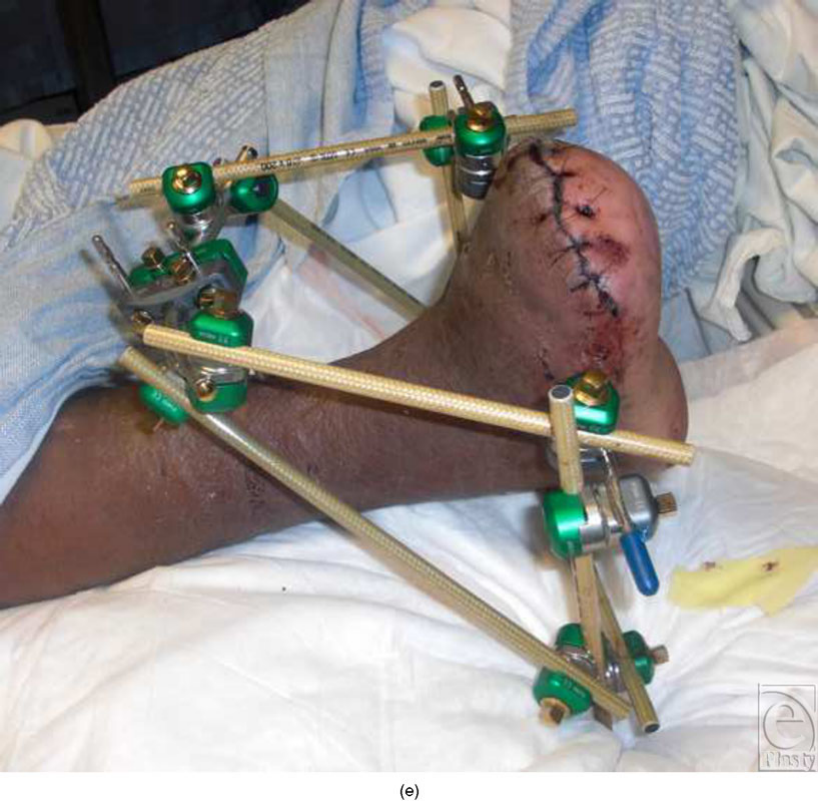

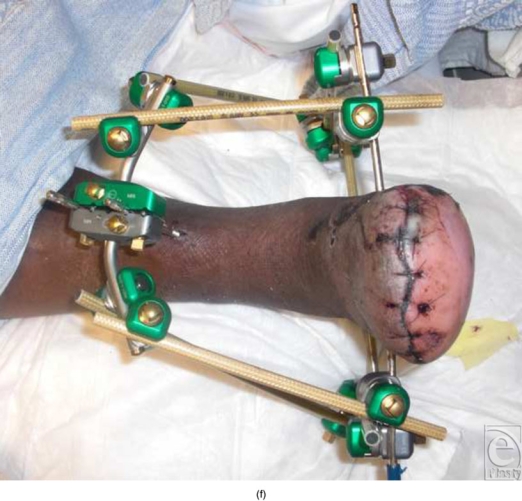
(a) Intraoperative photograph of the foot prior to revisional debridement and transmetatarsal amputation. (b) Plantar aspect of the foot after the completion of transmetatarsal amputation. (c) Application of half pins in the tibia and transfixion pin through the midfoot in the preparation for SALSAstand construction. (d) Immediate postoperative photograph after SALSAstand application. (e) Open nature of SALSAstand allows ease-of-dressing change and holds the foot in rectus position. (f) SALSAstand allows immediate or gradual triplanar correction of the foot in addition to offloading the heel.

A 62-year-old man with diabetes, peripheral arterial disease, morbid obesity, dialysis-dependent renal failure, and wet gangrene of the forefoot underwent percutaneous tibial revascularization for severe forefoot ischemia. Following tissue demarcation, a modified transmetatarsal amputation with rotational flap coverage was performed. After completing the transmetatarsal amputation, SALSAstand was applied and the patient's ankle joint was rotated and held in a 90° alignment in the frontal and sagittal planes after tendon balancing. The patient had no open heel wounds, but the device was applied as prophylaxis against the development of a heel decubitus and also to prevent subsequent tendon retraction and equinovarus deformity. The postoperative course was uneventful. SALSAstand did not require adjustment or removal because it allowed easy access for regularly performed forefoot dressings changes; the stand was removed in the clinic after 4 weeks (Fig 1).

### Case 2

**Figure 2:**
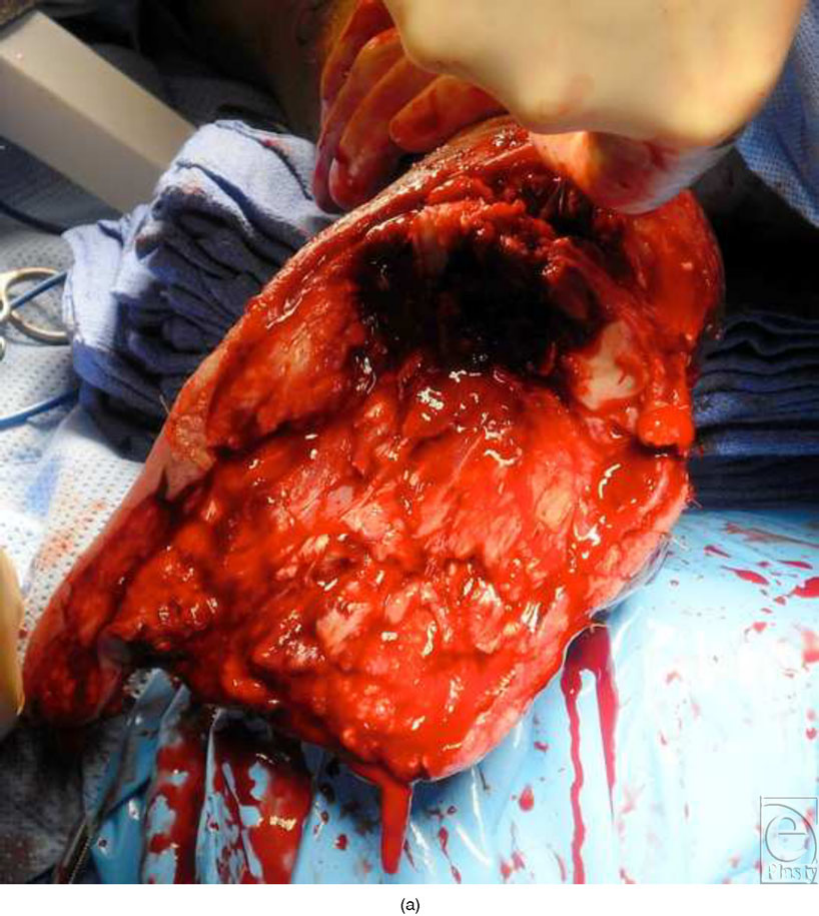

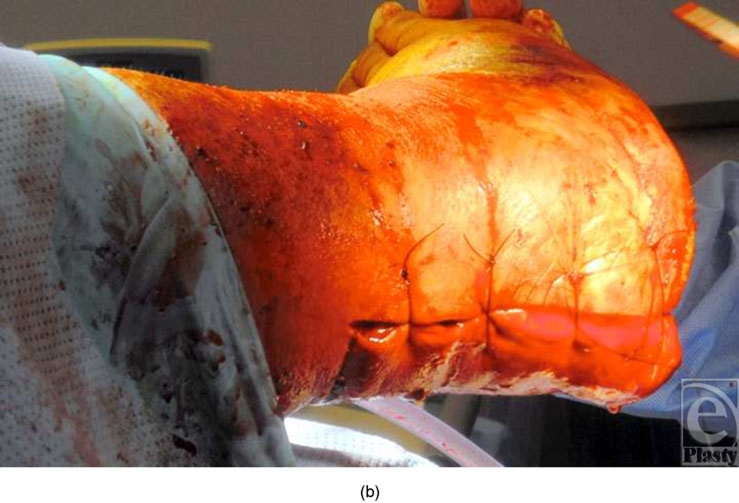

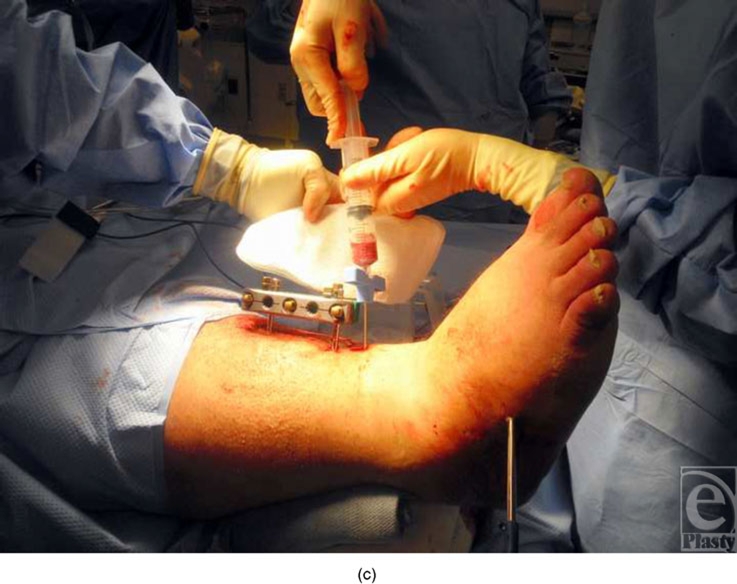

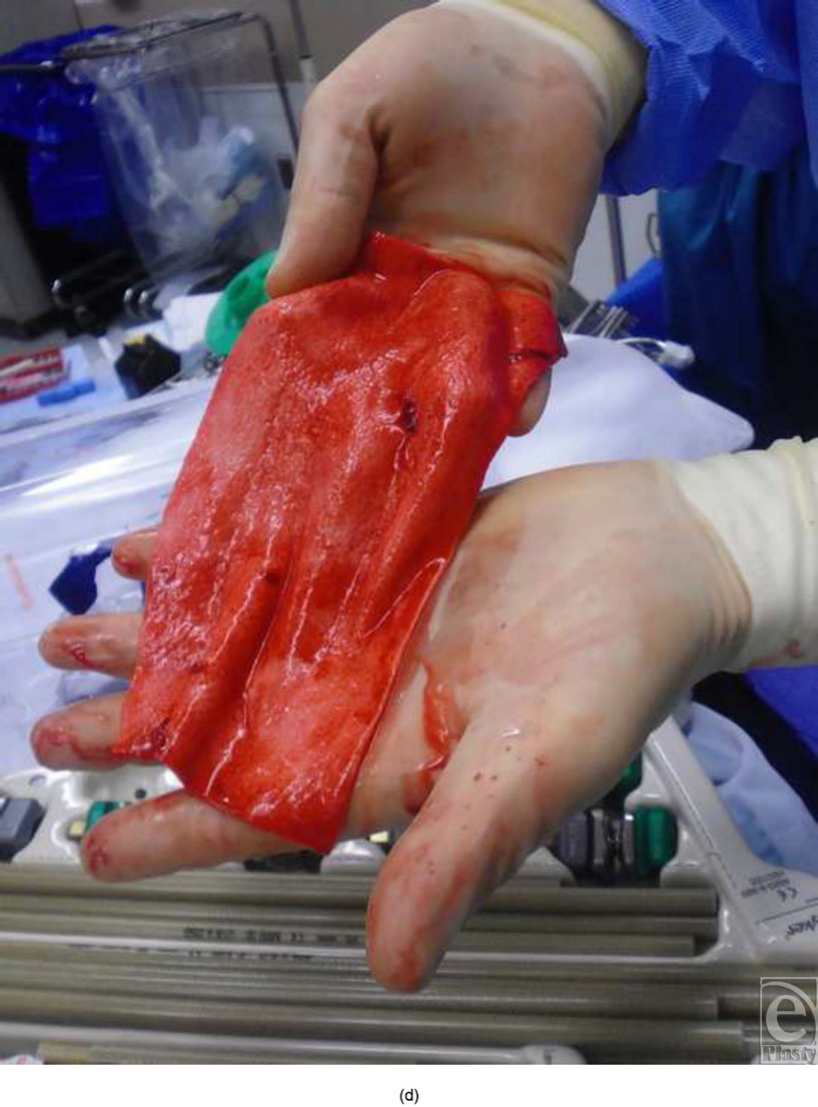

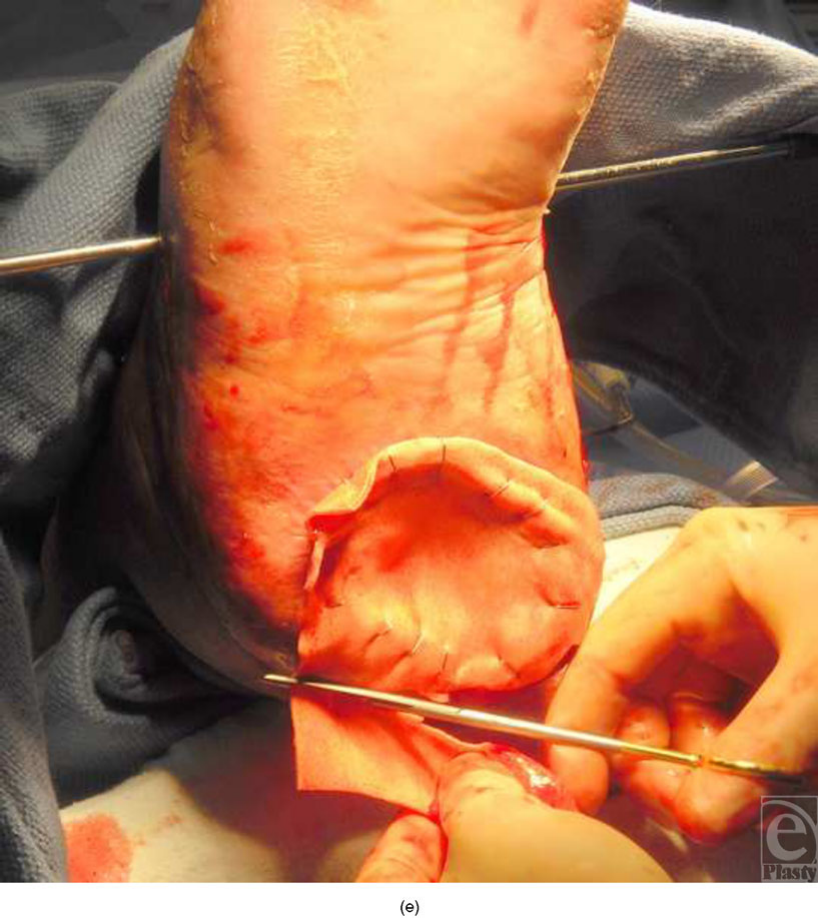

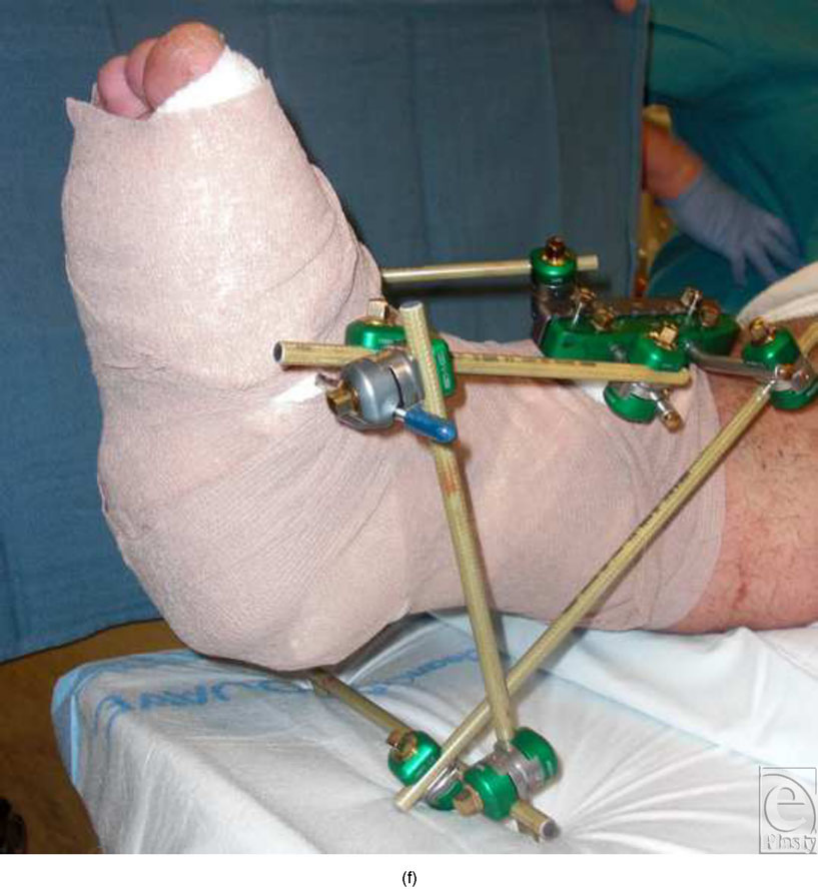
(a) Intraoperative photograph reveals a large, open, posterior heel wound after debridement. (b) Closure of posterior heel defect. (c) Bone marrow aspiration. (d) Preparation of acellular tissue matrix allograft. (e) Placement of acellular matrix. (f) SALSAstand construction around graft site demonstrating both posterior and plantar offloading of the posterior heel in addition to triplanar correction.

A 52-year-old morbidly obese man with diabetes and chronic, refractory plantar and posterior heel ulcerations underwent revisional calcaneal debridement with the placement of acellular matrix augmented by stem-cell seeding of the matrix with cells aspirated from the ipsilateral tibia. The open nature of the kickstand device allowed the placement of the matrix and care of the wound postoperatively, while effectively holding the patient in a corrected position and protecting the posterior aspect of the wound (Fig 2).

## DISCUSSION

This technique is a versatile tool deserving inclusion in the armamentarium of adjunctive procedures to reduce pressure on the posterior aspect of the foot because it allows immediate or gradual correction in 3 planes. Potential indications for this procedure include posterior foot wounds that require offloading, prevention of decubitus heel ulcerations, prevention of equinovarus deformity after partial foot amputation, and offloading of skin grafts or flaps. We also routinely employ this technique for stabilizing and protecting fragile flaps or grafts in high-risk patients in whom excessive motion about the foot could damage the graft. We have used this technique in various forms on more than a dozen patients. The rate of pin-tract infections has not been significantly different than what has been previously reported in the literature.

Relative contraindications include compromised soft tissue envelope that would result in pin placement across infected sites, severely poor bone stock that would compromise the strength of pin placement, those without sufficient vascular supply, and patients who are unable or unwilling to comply with the required postoperative period of non--weight-bearing status and immobility required of the affected part. These described contraindications are all relative because most patients on whom we have performed this procedure have had 1 or more of these comorbidities.
